# Pathologic Assessment of Rectal Carcinoma after Neoadjuvant Radio(chemo)therapy: Prognostic Implications

**DOI:** 10.1155/2015/574540

**Published:** 2015-10-05

**Authors:** Monirath Hav, Louis Libbrecht, Liesbeth Ferdinande, Karen Geboes, Piet Pattyn, Claude A. Cuvelier

**Affiliations:** ^1^Department of Pathology, Calmette Hospital, No. 3, Monivong Boulevard (93), Phnom Penh 12201, Cambodia; ^2^Department of Pathology, Ghent University Hospital, 9000 Gent, Belgium; ^3^Department of Gastrointestinal Oncology, Ghent University Hospital, 9000 Gent, Belgium; ^4^Department of Gastrointestinal Surgery, Ghent University Hospital, 9000 Gent, Belgium

## Abstract

Neoadjuvant radio(chemo)therapy is increasingly used in rectal cancer and induces a number of morphologic changes that affect prognostication after curative surgery, thereby creating new challenges for surgical pathologists, particularly in evaluating morphologic changes and tumour response to preoperative treatment. Surgical pathologists play an important role in determining the many facets of rectal carcinoma patient care after neoadjuvant treatment. These range from proper handling of macroscopic specimens to accurate microscopic evaluation of pathological features associated with patients' prognosis. This review presents the well-established pathological prognostic indicators and discusses challenging features in order to provide both surgical pathologists and treating physicians with a checklist that is useful in a neoadjuvant setting.

## 1. Introduction

Preoperative radiotherapy with or without chemotherapy (RCT) followed by total mesorectal excision (TME) has become a standard treatment for locally advanced rectal cancers [1–4]. The increasing use of RCT in rectal cancer creates new challenges for surgical pathologists, particularly in evaluating morphologic changes and tumour response to preoperative treatment. Various systems have been suggested for grading tumour response to RCT [5–10]. However, the majority of these systems do not consistently correlate with prognosis [11–14], and their reproducibility is poor [11, 15–17]. Moreover, RCT alters the macroscopic and microscopic assessment and prognostic relevance of a few well-recognized pathological features (i.e., tumour and nodal stage, circumferential resection margin, and lymphovascular invasion) [18–20]. For example, difficulty exists when no remaining tumours can be identified on macroscopic examination. In this context, accurate pathological tumour stage (ypT) depends on how assiduously the pathologists search for residual tumour, as well as on the number of blocks and slide sections processed. In addition, RCT may significantly decrease the number of retrieved lymph nodes [19]. This could cause underestimation of the nodal status in the absence of rigorous lymph node search. Controversy also persists concerning the optimal distal and circumferential margins [21–28]. Thus, surgical pathologists play an important role in determining the many facets of rectal carcinoma patient care after neoadjuvant treatment. These range from proper handling of macroscopic specimens to accurate microscopic evaluation of pathological features associated with patients' prognosis.

The aim of this review is to present the well-established pathological prognostic indicators and discuss challenging features, especially those with clinical impact, in order to provide a checklist that is useful in a neoadjuvant setting, for both surgical pathologists and treating physicians.

## 2. Macroscopic Assessment of TME Specimen

The TME technique is based on the sharp dissection of the avascular plane between autonomic nerve plexuses and the mesorectum. This operation results in the excision of the rectum enveloped by a mesorectal fat column of 2 to 3 cm. This part of the review presents the methods of assessment of mesorectum quality, circumferential resection margin (CRM), distal resection margin, and lymph nodes.

### 2.1. Evaluation of the Mesorectum

TME specimen handling begins with assessment of the quality of the mesorectum (Table 1) [29]. Inspection of the mesorectal surface gives the first indication of its quality. Full thickness slicing of the tumour and the mesorectum allows a good assessment of the adequacy of excision and the regularity of the CRM, which is the second indicator of the resection quality.

### 2.2. Specimen Processing

Quirke et al. [29] and Nagtegaal et al. [30] have developed an approach for the assessment and processing of the TME specimen. This assessment is performed by direct visual inspection of the fresh specimen; the anterior and posterior planes of the mesorectum are photographed to document their smoothness or irregularity. Then, the mesorectal fat is inked; care should be taken not to ink the peritonealized surfaces of the specimen, especially anteriorly, where the serosa extends lower down, as this may produce artifact and lead to difficulty in interpreting serosal involvement by upper rectal tumours that are either circumferential or anterior in their location [31]. Then, the specimen is measured and cut open along the anterior aspect from the top, leaving the bowel intact at a level just above the peritoneal reflection. After placing loose, formalin-soaked gauze wicks into the unopened segment of the rectum, the specimen should be pinned under tension on a corkboard to minimize shrinkage [32]. After an optimal fixation of at least 72 hours, the unopened segment is sliced transversely at 5 mm intervals in order to identify the area of deepest invasion, and the slices are photographed again to keep a record of the quality of the excised mesorectum, the tumour size, localization, and distance to all surgical margins. Concerning tumour sampling, the guideline of the Belgian Project on Cancer of the Rectum (PROCARE; http://www.kankerregister.org/), which was adapted from Quirke et al. [29] and Nagtegaal et al. [30], suggests that five initial blocks be taken from the site of the tumour or suspicious area. In cases with obvious macroscopic tumour remnant, in addition to taking the superficial and deepest part of the tumour, sections showing the closest relationship of tumour to CRM or to peritoneal surface as well as those containing the transition from suspicious mesorectal nodules to the CRM should be taken, as this allows proper evaluation of other pathological prognostic parameters [33]. When only mucin pools are found on gross examination, the entire suspicious zone must be sampled for accurate staging purpose, as residual viable tumour cells can be present upon microscopic examination in most cases [7]. Obviously, the pathologist should be informed about the precise location of the tumour before RCT in order to target tissue sampling effectively.

Recommended method for tumour sampling and examination in rectal carcinoma following radio(chemo)therapy and surgery is as follows.

**Step 1 .**

Take 5 blocks from the tumour or scarred area (assuming no obvious tumour was found grossly). These should include the superficial and deep part of the tumour as well as the relationship between the tumour and the CRM or peritoneal surface. If there are anymesorectal nodules, blocks containing their relation to the CRM should also be taken.

**Step 2 .**

If no viable microscopic tumour is identified within the initial 5 blocks, the entire scarred area should be sampled.

**Step 3 .**

If no residual tumour is found after examining sections from the additional blocks, three levels should be cut through each block. If no viable tumour cells are present even after rigorous examination of these sections, complete pathologic response or ypT0 can be reasonably and reliably reported.

### 2.3. Distal Margin

The distal margin, although less important than the CRM in terms of frequency of involvement and impact on recurrence, is still important to assess. There are two issues to keep in mind when considering the distal margin: the extent of intramural and extramural continuous tumour growth and the discontinuous distal mesorectal spread through lymphatics. In 20% of cases with positive nodes, there is lymphatic spread distal to the primary tumour. Furthermore, in many cases these positive distal nodes are located >2 cm away from the main tumour mass [34]. By contrast, intramural distal spread >2 cm is seen in only 3.6% of cases [35]. Zhao et al. [36] found that the rate of discontinuous tumour deposits within the distal mesorectum was 17.8% and that the extent of distal mesorectal spread was greater than the extent of intramural spread. From their data, they concluded that a 1.5 cm distal rectal wall margin and a 4 cm distal mesorectal margin are necessary to achieve adequate surgical clearance. Yet, in many cases, distal rectal wall margin of ≤1 cm also proved to be sufficient in preventing local recurrence, particularly in tumours limited to the rectal wall [37, 38].

One final issue to keep in mind, when measuring the distal margin, is shrinkage artifact. Goldstein et al. [39] have shown that a 5 cm length of colon and rectum in vivo is equivalent to 3 cm after resection and 2.2 cm after fixation. This highlights, once again, the importance of pinning the specimen on a corkboard to reduce the degree of shrinkage.

### 2.4. Lymph Node Retrieval

Lymph node status probably constitutes the single most important prognostic determinant in patients with rectal cancer whether treated preoperatively or not [40, 41]. International guidelines recommend that at least 12 lymph nodes are needed for adequate CRC staging [42, 43]. Nevertheless, there has been evidence suggesting that preoperative RCT for rectal cancer could reduce lymph node yield by roughly 33% [19, 44]. Despite this finding, a high motivation to retrieve as many nodes as possible must be maintained, since several studies support the concept that the more the nodes that are examined, the more accurate the staging. Moreover, the ratio of positive to total nodes retrieved, the so-called metastatic lymph node ratio, has been shown to be even more significantly associated with local recurrence and survival [45–47]. A number of adjunctive methods including alcohol treatment, xylene clearance, and ether-based methods have been developed in order to address the challenge of lymph node yield, but most of them require special equipment or the use of noxious volatile compounds [48–50]. Therefore, in many pathology laboratories, routine visual inspection, palpation, and dissection are still the standard of practice for lymph node retrieval, and meticulous examination, as well as the enthusiasm of the examiner, is one of the most important factors in determining the number of lymph nodes retrieved.

Once again, it is important to stress that the lateral mesorectal surface closest to the suspect nodes should be sampled together with the nodes to allow accurate evaluation of the CRM [33]. Thus, the orientation of suspicious perirectal nodules that are closely related to the CRM should be well preserved during the cut-up process, while the normally looking lymph nodes can be harvested in the usual manner, taking care not to overcount nodes that appear in more than one slice due to serial transverse slicing.

## 3. Microscopic Assessment

### 3.1. Tumour Histological Type

In the pathological reporting of colorectal cancer (CRC), the internationally accepted histological classification of colorectal carcinomas proposed by the World Health Organization [51] (WHO) is recommended by the College of American Pathologists (CAP) [52]. Based on this classification, the majority of rectal cancers are adenocarcinomas of no special type. Besides a few exceptions, histological type has no stage-independent prognostic significance [52]. The exceptions include the non-gland-forming tumours such as signet-ring cell carcinoma, small-cell carcinoma, and undifferentiated carcinoma, which are prognostically unfavorable [53–55], and medullary carcinoma, which is prognostically favorable [56]. In contrast to the findings in a few studies, mainly limited to univariate analyses, suggesting that mucinous adenocarcinoma may be an adverse prognostic factor [57–60], larger studies did not confirm mucinous histology to be a stage-independent predictor of poor outcome [61–66]. On the other hand, mucinous carcinoma tends to be prognostically favorable when associated with microsatellite instability (MSI) [67–69].

In summary, based on current evidence, it can be concluded that the only histological types of CRC that are prognostically significant are signet-ring cell and small-cell carcinoma (poor prognosis) and medullary and MSI-related mucinous carcinoma (favorable prognosis).

### 3.2. Tumour Differentiation Grade

In the WHO classification [70], grading of colorectal adenocarcinoma is based on the extent of gland formation. Therefore, the non-gland-forming histological types (e.g., signet-ring, small-cell, and undifferentiated carcinoma) are always regarded as high-grade or poorly differentiated tumours. In most cases, however, the estimation of the degree of glandular formation is subjective, resulting in interobserver variation, mainly in grading well and moderately differentiated tumours. The lack of uniformity in histopathological grading is further complicated by a number of different grading systems without consensus among pathologists [52, 71–73]. At present, the available data are insufficient to support one approach over the other, and the issue remains problematic. Irrespective of the complexity of the criteria, most systems stratify adenocarcinomas into four grades:

- Grade 1: well differentiated (>95% glandular formation),
- Grade 2: moderately differentiated (50%–95% glandular formation),
- Grade 3: poorly differentiated (5%–50% glandular formation),
- Grade 4: undifferentiated (<5% glandular formation).

Nevertheless, the most recent World Health Organization series on tumours of the digestive system recommends using the two-tier grading system (low versus high grade) in grading colorectal cancer [74]. Despite interobserver variation in assessment and the lack of standardization, histological grade has been repeatedly shown by multivariate analyses to be a stage-independent prognostic factor in a nonneoadjuvant setting [75–77]. After RCT, however, its impact on patient survival remains debatable [5, 11, 13, 78–80].

### 3.3. Lymphovascular and Perineural Invasion

The prognostic significance of lymphovascular (LVI) and perineural (PNI) invasion has been suggested and largely confirmed in a nonneoadjuvant setting [76, 77, 81–84]. Venous invasion has been demonstrated by numerous multivariate analyses to be an independent adverse prognostic factor in CRC [76, 77, 81, 82, 85–87]. In series of studies identifying the exact location of the involved vessels (i.e., extramural as opposed to intramural location), it was found that the extramural type was most predictive of survival [87–89]. In some studies, LVI, without distinction between venous and lymphatic vessels, has been found to be prognostically significant [81, 90]. More discordant results have been reported for lymphatic vessel invasion alone [91, 92]. It is likely that the disparities among existing studies on vascular invasion are related to inherent problems in the pathological identification of this feature. Definitive diagnosis of vascular invasion requires the identification of tumour within an endothelial-lined channel. However, this may be difficult when tumour induced vascular fibrosis or endothelial destruction is present. In addition, fixation artifact in the tumour may mimic small vessel involvement. For these reasons, interobserver variation may be substantial in the interpretation or recognition of vascular invasion. Additional limitations in the detection of vessel invasion are related to specimen sampling. For example, it has been shown that the reproducibility of detection of extramural venous invasion increases proportionally from 59% with examination of 2 blocks of tissue at the tumour periphery to 96% with examination of 5 blocks [89, 93, 94]. Other studies have suggested that taking various types of tissue blocks such as tangential ones in addition to perpendicular blocks raises the chances of detecting extramural venous invasion [94, 95].

Following preoperative RCT, the prognostic significance of LVI and PNI has been demonstrated in several studies, mostly by univariate analysis [96–98]. A study by Du et al. [97] showed that the disease progression of patients with LVI in irradiated tumours was significantly delayed as compared with that with LVI-positive tumours in nonirradiated tumours. They suggested that the aggressiveness of those tumour cells in the blood or lymphatic vessels may have been significantly weakened by radiotherapy, though they were not completely eliminated. In this respect, the stage-independent prognostic impact of LVI and PNI after RCT needs to be confirmed in larger studies with multivariate analysis.

### 3.4. Tumour Deposits (TDs)

Tumour deposits (TDs) are discrete adenocarcinoma nodules encountered in the pericolonic and perirectal fat during routine histopathological examination of advanced CRC specimens. Their prevalence in the mesorectum ranges from 6% to 64% [99–101]. TDs are histologically heterogeneous and may be associated with several types of recognizable anatomic structures such as veins, lymphatic vessels, and nerves, whereas in other cases carcinoma cells are seen scattered in small aggregates in the perirectal adipose tissue. This may account for the different classifications that TDs have undergone over time [102–104], particularly in the TNM classification series.  Table 2 summarizes the major changes in the last four editions of TNM classification for colorectal cancer.

The TNM5 introduced the 3 mm rule, resulting in a classification based exclusively on size, independent of histological features. Accordingly, discontinuous mesorectal tumour cell aggregates were considered as being primary tumour extensions (pT category) if measured ≤3 mm or as lymph node metastasis (pN category) if >3 mm [105].

The TNM6 replaced the size criterion with the shape criterion. Based on this classification, discontinuous mesorectal tumour nodules were considered as venous invasion if they have an irregular contour and as regional lymph node metastasis if they have the shape and smooth contour of a lymph node [106].

These two classifications have limited value since the TD classifications are based on a single morphologic criterion (i.e., size or shape). Moreover, the 3 mm rule was based on unpublished data, which were subsequently not confirmed [102, 107], and the shape criterion is insufficient to consistently distinguish different types of tumour involvement of the perivisceral fat [108], being the source of interobserver variation [107]. In 2009, Puppa et al. [103] proposed a new categorization of TDs:

- vascular-invasion type (extramural venous or lymphatic invasion): pT category,
- non-vascular-invasion type (smooth contour, surrounded by lymphocytes, not associated with veins or nerves): pN category,
- aggressive TDs (scattering pattern, not surrounded by lymphocytes, having close association with large vessels or neural invasion): pM1a (in-transit metastasis).

The TNM7 again introduced changes regarding the definition and classification of TDs. In the last edition, discrete foci of tumour found in the perivisceral fat or in adjacent mesentery away from the leading edge of the tumour and showing no evidence of residual lymph node tissue are considered to be TDs. If TDs are observed in lesions that would otherwise be classified as T1 or T2, then the primary tumour classification is not changed, but the nodule is classified in N1c category [109].

It seems that the different editions of TNM replace one subjective definition with another. Moreover, they do not appear to have prospectively tested this new TD classification and evaluated its reproducibility, since TNM7 states that a perivisceral nodule should be recorded as a positive lymph node if the nodule is considered by the pathologist to be a totally replaced lymph node (generally having a smooth contour) [109].

In summary, although the existing classifications of TDs need further improvement in terms of reproducibility and prognostic stratification, results from most studies on patients not receiving preoperative RCT indicated a worse prognosis for patients with TDs: increased local recurrence rates, increased development of distant metastases, and decreased survival [107]. In studies by Ratto et al. [99, 110] who looked at the incidence and prognostic impact of TDs in rectal cancer specimen after RCT, TDs occurred in up to 15.40% of cases and their presence was associated with reduced disease-free and overall survival. In contrast, Nagtegaal and Quirke [107] and Quirke et al. [111] considered the presence of TDs as a sign of good response to RCT. Whether or not the presence of TDs after RCT is a stage-independent predictor of poor outcome remains questionable. In daily practice, the presence of TDs must be included in the pathology report, specifying their total number, size, and growth patterns, in order to create more homogeneous groups of patients for enrolment in clinical trials [112].

### 3.5. Pathological Stage

Pathological staging following complete resection has long been considered the most powerful prognostic indicator in CRC [113]. The same holds true in rectal carcinoma after preoperative RCT [114–116]. Although a large number of staging systems have been developed for CRC over the years, the tumour node metastasis (TNM) staging system of the American Joint Cancer Committee (AJCC) and International Union Against Cancer (UICC) is by far the most widely recommended.  Table 2 lists the major changes made in the last four editions [109, 113, 117]. TNM 7th edition received a number of criticisms primarily for the new classification of TDs which lacks both scientific evidence and reproducibility [118, 119]. In reporting CRC, some centers prefer the 5th edition of the TNM classification to the other editions, mainly because of the reproducibility in TD classification [118, 120]. For future evaluation of the prognostic relevance of the changes in TNM classification, however, the 7th edition should be used, yet the conflicting feature, that is, TDs, should be reported in detail with description of their number, size, and growth pattern.

For accurate staging of treated rectal carcinoma, it is important to keep in mind that when microscopic remnants of tumour are not found, the scarred area must be entirely sampled [73, 120]. Moreover, if viable tumour is not present even after examining sections from the whole scarred area, three levels should be cut through each block to exclude residual tumour foci, as suggested in the CORE phase II study [121].

### 3.6. Acellular Mucin (aMUC)

In routine microscopic examination of neoadjuvantly treated rectal carcinoma specimens, mucus pools can be encountered in up to one-third of cases [122, 123]. With regard to this, a few recent studies have demonstrated that the presence of acellular mucin (aMUC) in rectal carcinoma after neoadjuvant RCT did not have significant impact on patient outcome [122, 124, 125]. de Campos-Lobato et al. looked at the prognostic value of aMUC in rectal cancer patients achieving ypT0 after preoperative RCT and concluded that aMUC did not affect local recurrence but may suggest a more aggressive tumour biology [125]. These findings are in support of the current CAP consensus statement that acellular mucin pools are not to be regarded as residual tumour and that their presence is to be recorded separately from the ypT category [122].

### 3.7. Local Inflammatory Response

**Figure 1:**
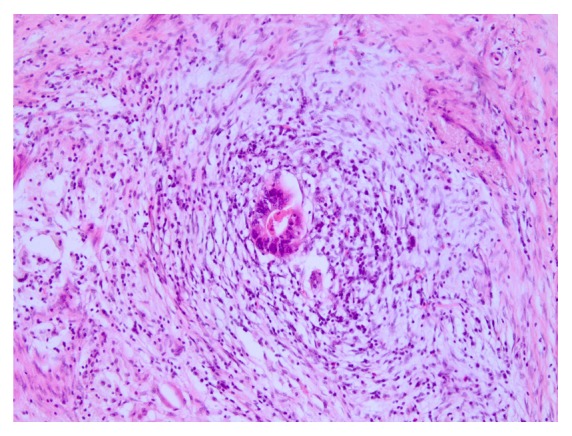
Pronounced fibroinflammatory changes after neoadjuvant RCT.

The tumour associated inflammatory infiltrate has long been considered a type of host response and an important prognostic factor in rectal cancer [126, 127]. After preoperative RCT, rectal cancer could undergo tumour regression by eradication of carcinoma cells and replacement by fibrous or fibroinflammatory tissues [123, 128, 129]. Nagtegaal et al. [130] and Shia et al. [123] found that patients with an extensive fibroinflammatory infiltrate around the tumour had lower recurrence rates. Two recent studies by Debucquoy et al. [128, 129] showed a better disease-free survival in rectal cancer patients whose TME specimens contained fibroinflammatory changes after RCT (Figure 1). Overall, a marked inflammatory cell component is not commonly seen in posttreatment rectal tumours [123, 128, 129]. Shia et al. [123] reported that, in 60% of cases, the inflammatory infiltration was only minimal. These findings imply an impaired or inhibited immune function in patients treated with RCT. Accordingly, it can be suggested that patients who maintain a more extensive inflammatory response at the tumour bed after RCT have a better outcome, and this factor is relevant in assessing the prognosis of these patients [123, 128, 129].

### 3.8. Therapy Response Assessment

Response to RCT ranges from minimal treatment effects to complete eradication of the primary tumour. Some authors used cellular-response grading which is based on the amount of residual viable tumour in relation to stromal fibrosis [5–8, 16, 131], whereas others looked at stage shift in the treated specimens, including tumour and nodal downstaging, when assessing response [11, 132, 133]. To date, none of the cellular-response grading systems has gained universal acceptance [11, 134], not only because the majority of them could not consistently predict patient outcome [11–14] but also because their reproducibility is poor, particularly for categories defining moderate to minimal regressive changes [11, 15–17]. On the other hand, evaluation of downstaging is objective and reproducible. Moreover, downstaging has been consistently demonstrated to correlate significantly with improved survival [11, 128, 129, 135]. Nevertheless, no study has investigated the prognostic impact of both cellular-response grading and downstaging in the same study cohort. Some studies [14, 136–138] specifically examined the prognostic impact of pathological complete response (pCR), defined by the complete absence of viable tumour cells in the primary tumour site (ypT0). The precise classification of pCR or ypT0 can be an effort- and time-consuming task provided that residual viable tumour cells could be identified in many cases upon rigorous microscopic examination (i.e., multilevel sectioning of the blocks containing the scarred area) [7, 121]. In this regard, the varying proportion of pCR observed in previous studies might have been due to the difference in dissection and examination methods used in each laboratory. In spite of this variation, the pCR status has been shown, in a few randomised trials and other studies, to significantly correlate with decreased local recurrence rate and improved survival [137–143].

### 3.9. Circumferential Resection Margin (CRM)

On microscopic examination, the distance of the tumour to the CRM may be the single most critical factor in predicting local recurrence (LR) after RCT and surgery [13, 29, 37]. The CRM involvement by tumour also has been shown to predict distant recurrence and overall survival (OS) in some studies [27, 144]. Although the definition of positive CRM varies among studies [27, 28], the majority of them involving several thousands of patients support the use of 1 mm as cut-off value for involved CRM [27]. The methods on which CRM measurement is based have been discussed in a study by Nagtegaal et al. [30] who looked at the difference in LR rates among cases with positive CRM as measured from the deepest point of invasion of the primary tumour and those with positive CRM as measured from invaded lymph nodes in the perirectal fat. They showed that patients with a positive CRM due to direct tumour extension developed local recurrence more frequently than those with a positive CRM due to positive nodes (22.1% versus 12.4%, *p* = 0.06). However, in the same study, there was no difference in the rate of local recurrence between patients with a positive CRM due to positive nodes compared to those with a negative CRM. As previously described, TDs are a frequent phenomenon in the mesorectum. Nevertheless, to date, no study has examined the prognostic relevance of CRM measurement based on the distance from lateral resection margin to TDs. Therefore, to allow further investigations on the prognostic significance of these two new CRM measurement methods (i.e., based on distance from positive nodes or TDs), it is recommended that, in cases with positive lymph nodes or TDs, practicing pathologists keep a record of the distance from the lateral resection margin to these perirectal tumour nodules in addition to reporting the classic CRM.

## 4. Summary and Conclusion

Preoperative RCT induces changes in both gross appearance of the surgical specimen and its pathological features, which could have impact on patient management and outcome. First of all, the assessment of the mesorectum is necessary as it is an important indicator of the resection quality. Then, the resected specimen should be sampled and examined properly, as summarized in Steps 1, 2, and 3, warranting not leaving out any prognostically relevant samples, particularly those containing the relationship of the lateral margins with the primary tumour and mesorectal nodules. Standardized protocols for the grossing of TME specimens should be available in order for pathologists, pathology residents, and pathologists' assistants to handle these specimens in a uniform and effective manner. Pathological features that have been consistently reported to significantly influence patient outcome after RCT include posttreatment pathological stage (ypTNM), microscopic status of the CRM, and local fibroinflammatory response, whereas the stage-independent prognostic value of histologic grade, LVI, PNI, and TDs requires further investigation in neoadjuvant setting. Concerning therapy response assessment, downstaging appears to be better than cellular-response grading in terms of both reproducibility and clinical outcome prediction.

## Figures and Tables

**Table 1:** Assessment of the quality of mesorectal excision or completeness of resection^*∗*^.

	Complete	Nearly complete	Incomplete
Mesorectum	Intact, smooth	Moderate bulk, irregular	Little bulk
Defects	Not deeper than 5 mm	Unexposed muscularis propria	Exposed muscularis propria
Coning	No coning	Moderate	Yes
CRM	Smooth, regular	Irregular	Irregular

CRM, circumferential resection margin.

^*∗*^Both the whole fresh specimen and formalin-fixed slices are examined to achieve optimal assessment.

**Table 2:** Major changes in the last 4 editions of TNM classification for colorectal cancer.

Edition (year)	T category	N category	M category	Stage grouping
4th (1987)	—	Introducing N3 category	—	—

5th (1997)	—	Removing N3 category	—	—
	TDs: introducing the 3 mm rule			—

6th (2002)	TDs: replacing the 3 mm rule with the contour rule		—	Subdividing stage III into IIIA, IIIB, and IIIC
	T4 split into T4a and T4b	ITC considered as N0		

7th (2009)	Changes in TD classification		M1 split into M1a and M1b	Subdividing stage IV into IVA and IVB
	—	Subdividing N1 into N1a, N1b, and N1c and N2 into N2a and N2b		

ITC, isolated tumour cells.
